# Age differences in experiencing meaning in life: A multidimensional approach

**DOI:** 10.1093/geroni/igab046.3272

**Published:** 2021-12-17

**Authors:** Nicole Long Ki Fung, Helene Fung

**Affiliations:** 1 The Chinese University of Hong Kong, Hong Kong, Hong Kong; 2 The Chinese University of Hong Kong, Shatin, N.T., Hong Kong, Hong Kong

## Abstract

Coherence, purpose and significance were defined as the three facets of the presence of meaning in life (Martela & Steger, 2016). This study investigated the age differences in the three facets of meaning in life between younger and older adults. We recruited 241 younger adults (Mage=18.72, SD=1.50, 36.5% male) and 114 older adults (Mage=64.93, SD= 5.94, 52.6% male) from Hong Kong to fill out a one-hour online survey. We adapted the Multidimensional Existential Meaning Scale, which measured sense of coherence, purpose, significance. In specific, the adapted version measured significance in two sub facets: the naturalistic significance (important to other people) and cosmic significance (impact lasts beyond lifetime and space). The results showed that older adults had higher sense of coherence (t=3.47, p<.001), higher sense of cosmic significance (t=6.29, p<.001) but lower sense of purpose than younger adults (t=-2.02, p<.05). There were no significant differences in naturalistic significance between the two age groups (t=1.24, p=.22). Within both age groups, participants had the highest score in purpose, followed by coherence and naturalistic significance. They had the lowest score in cosmic significance. This study illustrated that younger and older adults have different absolute levels of meaning facets but are similar in the relative levels of meaning facets. Further studies can investigate how changes in absolute levels of meaning facet and preservation of the relative levels may affect well-being across age.

